# A Case of Hernia, in Which the Operation of Taxis Was Rendered Efficient by a New Mode of Performing It

**Published:** 1824-11

**Authors:** William Balfour

**Affiliations:** the University of Edinburgh.


Art. VI.—*A Case of Hernia, in which the Operation of Taxis was rendered efficient by a new Mode of performing it.* By William Balfour, m.d. of the University of Edinburgh.

Certain general principles, the result of accumulated observations and experience, are necessary to direct the physician and surgeon in the application of the means he employs in the cure of every case of importance that comes under his care. Without such aid, no individual mind could embrace, no individual could retain, the innumerable forms in which disease presents itself, and the consequent variety of remedies, and necessarily varied modes of applying them. Necessary, however, as theory is in the practice of the healing art, sufficient scope is left to the genius, the resources, and discretion, of every practitioner. No set of rules will apply in every instance, and cases but too frequently occur, in which the ordinary routine is altogether inefficient. In such emergencies, if experiments can be made which promise some advantage, without increasing the risk of the patient, it is the duty of the practitioner to make them; and, if productive of the desired effect, they add new facts to the stock of human knowledge. It is in this way alone that an experimental science can be advanced;—it is in this way that the practice of medicine and surgery has been improved.

The rules laid down by authors for conducting the operation of taxis, are not precisely the same. The difference, however, between the latest and best writers is more in appearance than in principle; complete relaxation of the parts affecting the aperture through which the bowels protrude, being the object of all the variety of directions for position of the body.

Winslow directs the body to be placed on an inclined plane, the thigh being bent towards the trunk. Sir Astley Cooper advises the same. Mr. Samuel Cooper directs the thorax to be elevated, and turned towards the opposite side, and the thigh to be bent and rotated inwards. Mr. Hey says, "the patient should lie on the side opposite to that affected by the hernia; the abdominal muscles being relaxed by bending forwards, and the thigh brought to a right angle with the trunk." Mr. Benjamin Bell recommends a mode of proceeding very different from any of those now quoted: he says, "the patient should be placed almost perpendicularly upon his head;" and that placing the patient's feet over the shoulders of another person, while at the same time his body is allowed to hang downwards, and causing him in this posture to be a good deal jolted about, has on some occasions been known to answer, when every other means has been tried in vain. "This position," says Mr. Bell "causes almost the whole quantity of intestines to hang or swing by the protruded parts." And a most efficient mode this would be, were it at all practicable; but if relaxation of the femoral fascia, of Poupart's ligament, of the abdominal muscles and ring, of the internal iliac and psoæ muscles, facilitates the reduction of femoral and inguinal hernia, then Mr. Bell's method must be the very worst that can be adopted. In the posture he recommends, these parts must be more upon the stretch than when the patient stands upright upon his feet. "Suspension," says Mr. Hey, "over the shoulders of an assistant or two has been thought to favour the reduction considerably. I have tried it often; but, having found it inefficacious, I have now for many years laid it aside."

Taxis consists in placing the body in a proper posture, and applying gentle pressure to the tumor, in the direction in which its contents protruded.

In the month of August, 1814, I was called to Mr. Samuel Malcolm, aged twenty-eight, whom I found labouring under symptoms of strangulated scrotal hernia. He had worn a truss for several years, but, the strap having broke, had laid it aside for two days previous to that on which I was called to him; when a hernial tumor, of considerable size, was formed, and refused to recede by the means he usually employed on similar occasions. I found him in bed, on account of a chilliness of which he complained, and general tenderness of the tumor to the touch; pulse natural.

I put my patient in the posture recommended by Mr. Samuel Cooper, and then proceeded according to Mr. Hey's directions, "by gently compressing the neck of the hernial sac, that its bulk might be diminished where the stricture was the greatest; and then pressing the diminished part towards the abdomen." I continued my exertions, with short intervals, for fifteen or twenty minutes, without effect. The patient had now become thirsty, yawned incessantly; and the tenderness of the parts had increased so, that he intreated me to desist.

Finding I could not succeed, and satisfied that the tumor was exclusively intestinal, it occurred to me that, if I could pull the protruded parts downwards a little, inflammation would be retarded, by the constriction being removed to another portion of the bowels; that both constricting and constricted surfaces would in some degree be lubricated; that the distention of the vessel of the incarcerated portion of the gut would be taken off, and consequently its whole bulk diminished; and that the peristaltic motion might be restored: effects, which would all conspire in facilitating reduction.

But is there no injury to be apprehended, I asked myself, from using such freedom with the parts? If resistance, I replied, is considerable, I will desist: if there is little or no obstruction to their further descent, I can do no harm; for inflammation, however imminent, has not yet commenced. Besides, if Mr. Benjamin Bell and Mr. Hey could suspend the whole weight of the intestines by the protruded part without injury, much less risk must be run in applying a power which can be regulated at pleasure. Supporting the scrotum, therefore, in my right hand, and doubling up the integuments with the finger and thumb of my left, I laid hold of the gut, and pulled it gently downwards. Immediately on feeling it yield, I applied pressure upwards as before; when the tumor receded into the abdomen in the pleasantest manner, and with a gurgling noise.

From the date it will be seen that the above case occurred ten years ago. Indeed, it had entirely escaped my memory, till, rummaging one day among a parcel of manuscripts, it presented itself to my view. I now lost no time in taking the opinion of some of the most eminent practitioners in this city on the subject. Every one stared at mention of the fact, as if he would have said, "How simple the idea! It is astonishing it did not occur to myself." All agreed that it was new, and that it unquestionably superseded the necessity of having recourse to the knife in the present instance. Now, any mode of cure that succeeds in one case may succeed in others similar to it.

As illustrative of the probable advantage to be derived from the fact in question, I would suggest a consideration of the phenomena which primarily occur in hernia. What, for instance, must be the immediate effect of even the slightest circular compression of the intestinal tube? Proportionate distention, certainly, of the vessels below the stricture, or of the isolated portion of the gut. It is very conceivable that cases of hernia frequently occur, in which the protruded parts completely fill the aperture through which they pass, without being sensibly constricted for a considerable length of time; and yet distention of the vessels below the aperture gradually, though imperceptibly, advances to that pitch at which it forms an insurmountable obstacle to the retrocession of the gut. A very slight degree of such distention may effectually preclude success in the operation of taxis. But if, in such circumstances, the stricture can be shifted, as in the above case, to a different portion of the tube, the necessary consequence would be a diminution of distention of the vessels of the isolated portion. If to these are added the other considerations which determined my practice in the present case, they will form a powerful *a priori* argument for its adoption in many cases of daily occurrence. At the same time, I am well aware that the utility of any new fact or practice in medicine and surgery can only be determined by time and experience.

Among others of my professional friends here, I submitted this case to Mr. Liston, surgeon, who, in a letter to me on the subject, was pleased to express himself in the following terms:

"My dear Sir,—I have read your paper on Hernia with great pleasure. The preliminary remarks appear to me most admirable and judicious. The practice is certainly new, and founded on sound principles; but I should doubt the practicability in some instances, and the propriety of it in others. When the prolapsus is recent and the coverings lax, it may prove a very useful proceeding; but experience [an iteration of my own sentiment] must determine the cases in which it is likely to prove beneficial and safe. The doing away with a painful operation is at all times laudable; but, in spite of all our endeavours, I suspect cases will occur in which the knife, early resorted to, will afford the surest and safest relief to the patient."

Thus, Mr. Liston's opinion is, on the whole, favourable to the practice in question. He has his *buts* and his *doubts*, to be sure; which are equally applicable to taxis as usually performed, as with the addition which I made to it in the preceding case. That it may prove impracticable in some instances, and improper in others, may be affirmed, indeed, of any remedy in any disease whatever. General blood-letting may be imperatively indicated in the commencement of fever, but would prove certain destruction in the latter stages.

Mr. Liston admits that "the practice is certainly new, and founded on sound principles;" and that, "when the prolapsus is recent, and the coverings lax, it may prove a very useful proceeding." This is all I contend for,—and it is a great deal: I never dreamt of an alternative to the knife in the advanced stages of incarcerated hernia. At the same time, I think it perfectly logical to affirm that, if the step I have added to the operation of taxis shall be found, even occasionally, to facilitate or render that operation more efficacious, then it may supersede, to a proportionate extent, the necessity of a painful operation, by enabling the practitioner to remove the complaint before it has advanced to that stage in which the knife alone can be of avail to the patient.

*Edinburgh; 12th August*, 1824.

